# Early Postnatal Exposure to Intermittent Hypercapnic Hypoxia (IHH), but Not Nicotine, Decreases Reelin in the Young Piglet Hippocampus

**DOI:** 10.1007/s12640-022-00598-0

**Published:** 2022-11-02

**Authors:** Vanessa Despotovski, Arunnjah Vivekanandarajah, Karen A. Waters, Rita Machaalani

**Affiliations:** 1 Discipline of Science, Life and Environmental Science, The University of Sydney, Camperdown, NSW Australia; 2 Discipline of Medicine, Central Clinical School, Children’s Hospital at Westmead Clinical School, Faculty of Medicine and Health, The University of Sydney, Camperdown, NSW Australia; 3 Discipline of Child and Adolescent Health, Children’s Hospital at Westmead Clinical School, Faculty of Medicine and Health, The University of Sydney, Camperdown, NSW Australia

**Keywords:** Dentate gyrus, Dorsal, Ventral, Hippocampal formation, Granule cell dispersion, SIDS

## Abstract

This study evaluated the expression of reelin, an extracellular protein involved in lamination and migration of neurons, in the hippocampus of young piglets, and quantified to examine the following: (i) baseline levels within layers of the hippocampus and dentate gyrus (DG); (ii) differences between ventral and dorsal hippocampi; and (iii) changes attributable to postnatal exposure to continuous nicotine for 12 days, or intermittent hypercapnic hypoxia (IHH), with further analysis according to duration of IHH (1 vs 4 days). Additionally, we analysed whether any exposure altered DG morphology and whether it is related to altered reelin expression. Reelin was visualised via immunohistochemistry, and the number of positive reelin cells/mm^2^ was measured in the CA4/Hilus, layers of the DG, and the CA1. The dorsal DG had significantly more reelin within the subgranular zone compared to the ventral DG (*p* < 0.01). There was no difference in reelin between nicotine (*n* = 5) and controls (*n* = 5). IHH exposed piglets (*n* = 10) had significantly lowered reelin in the CA1 (*p* = 0.05), specifically the stratum pyramidale (*p* = 0.04) and the hippocampal fissure (*p* = 0.02), compared to their controls (*n* = 7); the duration of IHH had no effect. No exposure was associated with an alteration in DG morphology. This study shows that postnatal IHH exposure decreased reelin expression in the developing piglet hippocampal CA1, suggesting that IHH may result in altered neuronal migration.

**Supplementary Information:**

The online version contains supplementary material available at 10.1007/s12640-022-00598-0.

## Introduction

The migration and formation of granule cells in the brain is a process that is vulnerable to insults during early development, whether these are experienced prenatally (reviewed in Nalivaeva et al. 2018) or postnatally (Rice and Barone 2000). A commonly studied insult is hypoxia, for example, due to pregnancy related factors such as preeclampsia, hypoxic ischemic encephalopathy at birth, or cardiorespiratory factors postnatally (reviewed in Nalivaeva et al. 2018). When present, hypoxia alters levels of proteins that are critical for neurogenesis (Golan et al. 2009), and genes involved in neuroplasticity (Howell and Pillai 2016).

A brain region where both neurogenesis and plasticity are very active during this period of development is the hippocampus, with sub-regions being the dentate gyrus (DG) and Cornu ammonis (CA) 1–4 (CA4 often also known as the Hilus). Reelin, an extracellular matrix protein secreted by Cajal-Retzius (CR) cells, is critically involved in the cellular organisation and development of the hippocampus and DG (Frotscher 1998; Folsom and Fatemi 2013), and more specifically involved in synaptic plasticity (Roberts et al. 2005; Qiu et al. 2006). Alteration of reelin expression is thought to underlie altered morphology of the granule cell layer of the DG, whereby decreased reelin results in granule cell dispersion (GCD) (Heinrich et al. 2006).

Most studies examining the effects of hypoxia on reelin expression have focused on the cerebral cortex and cerebellum, as they are designed to inform the clinical conditions of perinatal asphyxia (Haramati et al. 2010; Vázquez-Borsetti et al. 2019) and neuropsychological abnormalities (Komitova et al. 2013; Howell and Pillai 2014, 2016). Hypoxic insults have been associated with decreased reelin expression (Haramati et al. 2010; Komitova et al. 2013; Howell and Pillai 2014, 2016; Nisimov et al. 2018; Zhang et al. 2021), with the exception of one study where an increase was found in the prefrontal cortex and in layers I and II of the anterior insular cortex, although in that study expression was decreased in layer VI (Vázquez-Borsetti et al. 2019), thus raising the possibility of region-specific sensitivities to hypoxia. Studies of the hippocampus using tissue homogenates (Golan et al. 2009; Howell and Pillai 2014) also reported a decrease in reelin expression in response to hypoxia, whereas a study of neonatal hypoxia-ischemic injury focused on the DG showed increased reelin at postnatal ages 14 and 21 days (Zhang et al. 2021). To our knowledge, no previous study has examined postnatal effects of a hypoxic insult on reelin expression, with attention to regional specificity on all the different layers of the hippocampus and DG during infancy.

Another insult of interest to us is nicotine exposure given its role in inducing abnormal cardiorespiration, particularly during early infancy (Vivekanandarajah et al. 2019). Studies on the effects of nicotine on reelin expression in the hippocampus are scarce (Romano et al. 2013a, b; Ohishi et al. 2014) with results indicating no fluctuation of reelin expression in rodents treated with nicotine, but these studies were limited to the Hilus of the DG (Ohishi et al. 2014) or homogenates of the whole hippocampus studying reelin mRNA via PCR (Romano et al. 2013a, b; Ohishi et al. 2014).

Our laboratory has developed and studied two piglet models during infancy: one of intermittent hypercapnic hypoxia (IHH) (Waters and Tinworth 2001) and the other of continuous nicotine infusion (Machaalani et al. 2005). The IHH model was designed to mimic the gaseous exchange present when infants sleep prone, bedshare, or suffer from obstructive sleep apnea (OSA), three factors implicated in increasing the risk for sudden infant death syndrome (SIDS). Within this model, the duration of IHH was further analysed based on the clinical context that some SIDS infants may have experienced an acute IHH exposure on the night of death when found prone for the first time (Côté et al. 1999) vs those exposed to repetitive IHH, as with bedsharing. The continuous nicotine infusion model was designed to mimic postnatal cigarette smoke exposure where nicotine is a main neuromodulating constituent of cigarettes (reviewed in Valentine and Sofuoglu 2018). This study, through immunohistochemistry, examined the DG and CA1 layers of the hippocampus from these piglet models to determine if these insults have the ability to alter reelin expression, and whether they are associated with morphological abnormalities, including GCD. Our focus was on the ventral hippocampus, although sub-analysis of the dorsal hippocampus was also undertaken to determine if any differences exist supporting the functional differences reported whereby the ventral hippocampus is generally associated with stress, emotion, and behaviour while the dorsal is associated with navigation, learning and memory (Fanselow and Dong 2010). Based on available literature, we hypothesised that nicotine would have no significant effect, while IHH would be associated with decreased reelin expression.

## Methods

### Animal Model and Brain Tissue collection

For the current study, we included 2 groups with IHH exposure: one of an acute 1 day exposure (1D IHH) and another of repeated exposure over 4 consecutive days (4D IHH). The methodology of the live piglet work was previously detailed in Waters and Tinworth (2001). Briefly, non-sedated piglets aged 10–13 days were placed into a temperature-regulated perspex box, harnessed into a vinyl hammock, and a full-face nasal mask was sealed around their snout, through which piglets breathed either exposed an air mixture or HH gas mixture (8% O_2_, 7% CO_2_, balance N_2_). Piglets were alternately subjected to 6 min of either exposure for a total of 48 min per day. Controls were placed in the same experimental conditions but only exposed to air for a 1- or 4-day period (Waters and Tinworth 2001) and we refer to them as 1D air and 4D air, respectively.

Regarding nicotine exposure, this was previously detailed in Machaalani et al. (2005). Piglets aged 1–2 days underwent aseptic surgery for intraperitoneal insertion of an osmotic minipump (Alzet; Alza Corporation, USA, Model 2ML2). Nicotine was delivered as a nicotine (-) hydrogen tartrate salt at a dose of 2.0 mg/kg/day dissolved in sterile water for 12 continuous days. The control group pump delivered sterile water only. To prevent cross-contamination of nicotine, piglets were housed separately. To confirm that the nicotine exposure equated to the same levels reported in babies exposed from cigarette smoking households, cotinine, a metabolite of nicotine, was measured in blood and urine samples collected at the time of euthanasia, and comparable levels were found (Machaalani et al. 2005).

All piglets were euthanized at the age of 13–14 days, through an overdose of pentobarbital (200 mg/kg piglet body weight), the whole brain removed, and immersion fixed in 10% neutral buffered formalin for 14 days. Regions of interest from the left hemisphere were separated and sectioned into 4-mm slices and returned to the 10% neutral buffered formalin for another 5 days, before being processed to paraffin (Machaalani et al. 2005).

The hippocampus block was obtained in the coronal plane and was sectioned at 7 µm by a rotary microtome (Leica RM125, Leica Biosystems Pty LTD, VIC, Australia), with sections mounted onto salinized microscopic slides, dried overnight at 37 °C, and stored at room temperature for a minimum of 1 week before staining.

### Immunohistochemistry

Immunohistochemistry (IHC) was applied using a standard protocol from our laboratory (Machaalani and Waters 2003) and detailed in Despotovski et al. (2021). Given the number of tissue sections to be stained, not all were able to be added in the same IHC run. Three cases were thus used to determine reproducibility of two sections stained within the same run (intra-assay) or over two different runs (inter-assay) on quantification, and found to be within 5%. Regardless, all the nicotine and saline cases were stained within the same run, while those of the IHH groups were stained over two runs.

All steps were conducted at room temperature unless otherwise indicated. Briefly, after de-paraffinisation in xylene and rehydration through a graded series of ethanol to distilled H_2_O, tissue sections underwent antigen retrieval by microwaving on high in TRIS–EDTA buffer (1 Mm EDTA, 1 Mm sodium citrate, 2 mM Tris, pH 9.0) for 14 min. Endogenous peroxidase activity was blocked by incubating sections in 50% methanol, 50% phosphate buffered saline (PBS), and 3% hydrogen peroxide (H202) for 20 min. To avoid non-specific staining, sections were blocked using 10% normal horse serum (NHS) for 30 min and then incubated with the primary antibody reelin (Mouse monoclonal (MAB5366), Millipore, 1:1000 dilution in 1% NHS) overnight. Sections were washed with PBS and incubated with biotinylated secondary anti-rabbit/anti-mouse IgG made in horse (BA-1400, Vector Laboratories, 1:300 dilution in 1% NHS) for 45 min and then with avidin–biotin complex (ABC, PK-4000, Vector Laboratories Inc.) for 45 min. Reaction was visualised via incubation with 3,3’ diaminobenzidine tetrachloride (DAB, SK-4100, Vector Laboratories Inc.) for a maximum of 8 min, and counterstained with Harris Haematoxylin. Sections were then dehydrated through graded ethanol to xylene, mounted and cover slipped with dibutylphthalate polystyrene xylene (DPX).

### Image Capture and Quantitative Analysis

All hippocampal sections were captured at × 20 using a slide scanner (AxioScanZ.1, Carl Zeiss Microscopy; Jena, Germany) and a single section per case was imaged and quantified using the supporting software (Olympus OlyVIA 3.2).

**Fig. 1:**
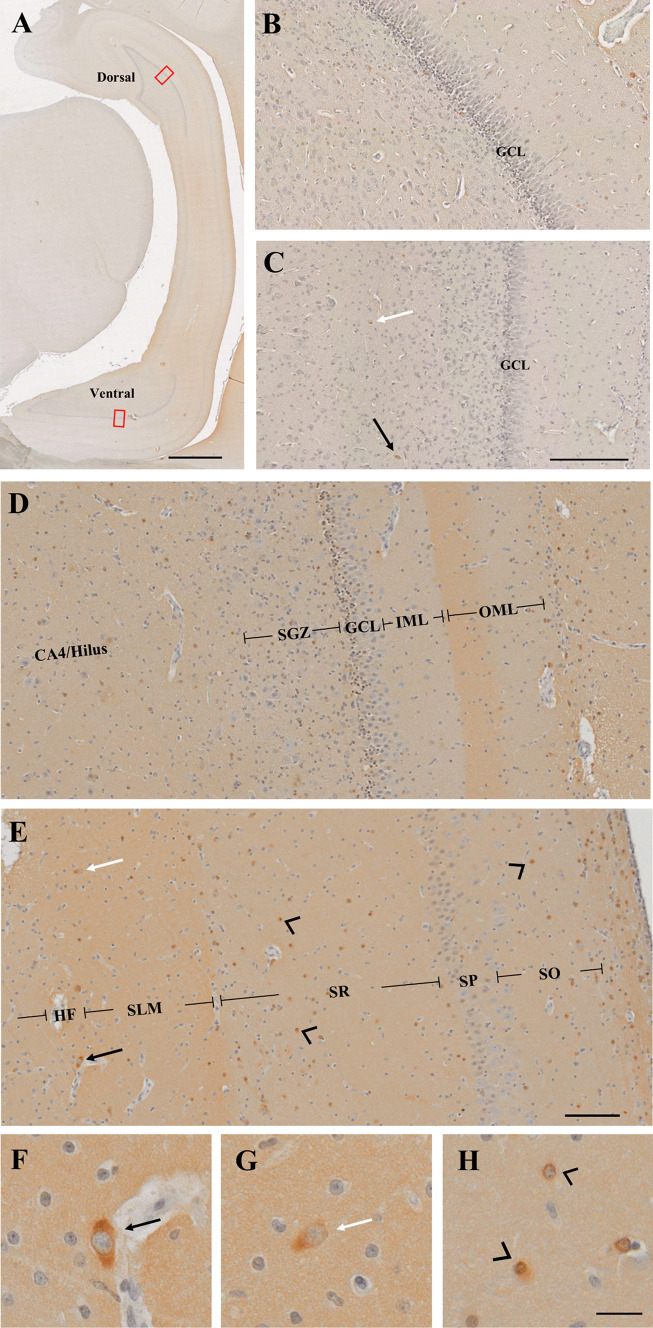
Reelin staining in the hippocampus and its layers. **A** Cross-section of the hippocampus where both the dorsal and ventral hippocampi are present (estimated as 20.0 mm from the rostral horn of the lateral ventricle (Guidi et al. 2011)). Red boxes indicate the areas quantified and are magnified in **B** dorsal DG and **C** ventral DG. Boundaries of the layers quantified are shown for **D** the ventral DG and **E** ventral CA1. Magnification of positive cells being **F** bipolar, **G** unipolar, and **H** circular. Black arrows = bipolar cells, white arrows = unipolar, black arrow heads = circular. GCL, granule cell layer; IML, inner molecular layer; OML, outer molecular layer; SO, stratum oriens; SP, stratum pyramidale; SR, stratum radiatum; SLM, stratum lacunosum moleculare; HF, hippocampal fissure. Scale bar **A** = 2 mm, **B**–**C** = 200 µm, **D**–**E** = 100 µm, **F**–**H** = 20 µm

**Fig. 2:**
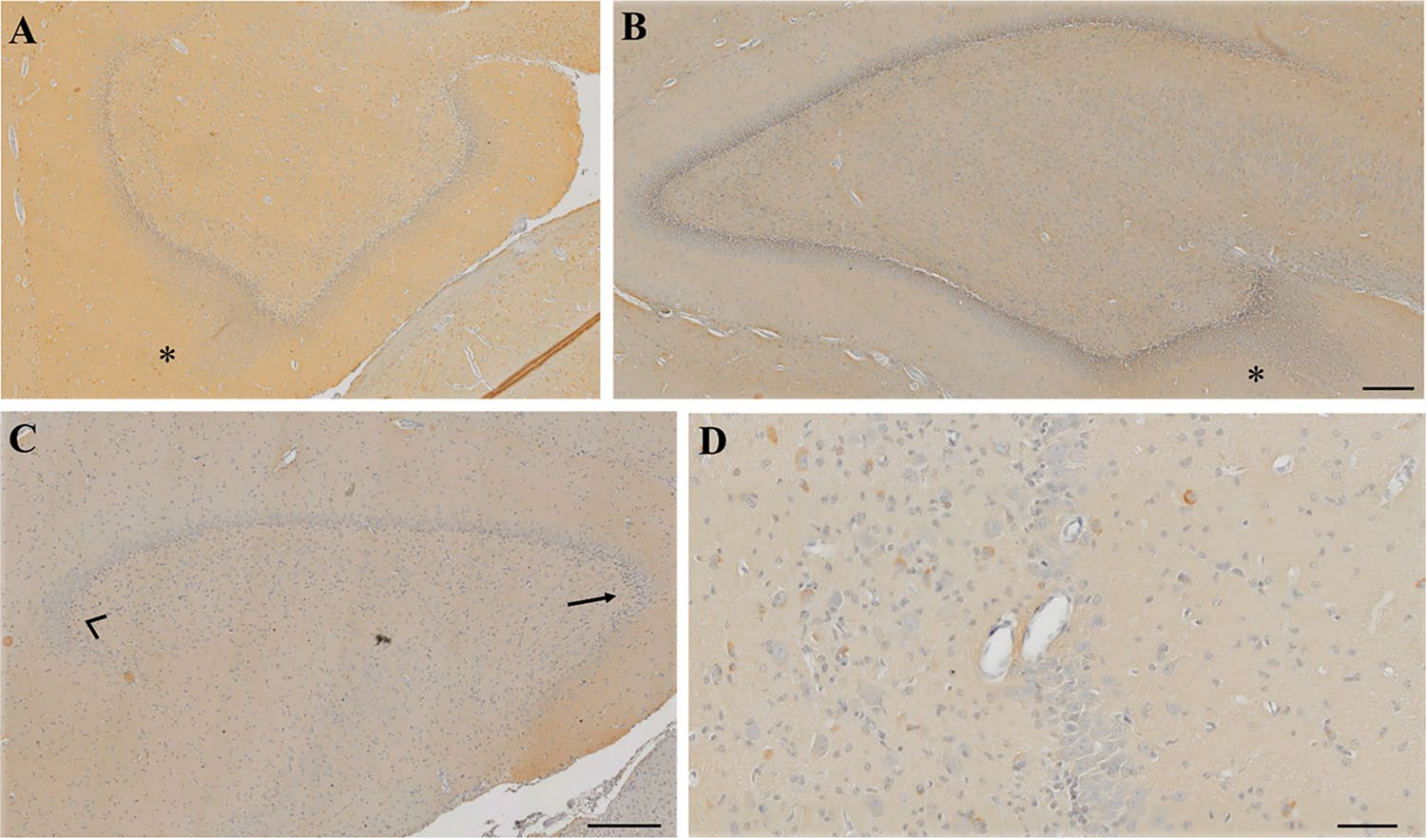
Reelin-stained sections showing GCD in the dorsal DG of **A** saline and **B** nicotine exposed piglets. GCD is shown by the asterisk (*). **C** illustrates mature DG cells in the GCL (based on size and shape) on the external limb (arrowhead) compared to immature cells (arrow) on the internal limb. **D** A blood vessel separating the dentate GC’s in the GCL with no reelin in the immediate vicinity. Scale bar **A**–**C** = 200 µm, **D** = 50 µm

After sub-group analysis to examine for differences in reelin expression in ventral and dorsal hippocampal structures (Fig. 1A), the focus of this study was on the ventral hippocampus given that the majority of our tissue had this present. Our tissue is estimated to be between 15.4 and 20.0 mm from the rostral horn of the lateral ventricle (Fig. 2; (Guidi et al. 2011)).

The quantitative method we applied was the one we applied recently in our human study determining the effects of aging on reelin in the hippocampus (Despotovski et al. 2021). This method is also consistent with other animal model studies (Abraham and Meyer 2003) representing the data as the number of positive reelin cells/mm^2^ per region analysed. This analysis was undertaken using a set box and quantifying 1–2 fields per layer, rather than the whole layer, based on a pilot study on two tissue sections which showed within 20% variability amongst the fields of the whole hippocampal formation which equated to 10–15 boxes pending on the size of the formation from one piglet to another.

The straight limb of the ventral DG (Fig. 1A) was identified, and a box with an area of 0.3mm^2^ was drawn encompassing the following layers: Cornu ammonis/Hilus (CA4/Hilus), subgranular zone (SGZ), granule cell layer (GCL), inner molecular layer (IML), and the outer molecular layer (OML). Positive reelin cells were counted within their respective layers. The ventral DG was counted at two separate locations along the straight limb and averaged. Adjacent to the SGZ of the DG, the CA4/Hilus was quantified separately. A box was drawn directly underneath the SGZ to determine if there is any significance with reelin expression in neighbouring layers and overall neurogenesis of the DG.

Next, the CA1 was differentiated by its cell type and a box with an area of 0.5mm^2^ was drawn including the layers of stratum oriens (SO), stratum pyramidale (SP), stratum radiatum (SR), stratum lacunosum-moleculare (SLM), and hippocampal fissure (HF). Positive reelin cells were counted within their respective layers.

A total of 6 cases (*n* = 3 saline controls, *n* = 3 nicotine exposed) had both the dorsal and ventral hippocampus within the same section. In these cases, the DG and CA4/Hilus were quantified except that due to the differing DG structures and size, only one boxed area of 0.3mm^2^ of the dorsal DG was quantified.

The following morphological features of the DG (Blümcke et al. 2009; Kinney et al. 2015; Machaalani et al. 2021) and CA1 (Kinney et al. 2015) were sought on microscopic examination: GCD, focal granule cell bilamination, blood vessels in the GCL and SP, and gaps in the SP layer. Our terminology of external and internal limbs of the DG were applied as defined by Blümcke et al. (2009) and Machaalani et al. (2021).

### Statistical Analysis

Raw counts and areas were collated in Microsoft Excel, converted to positive reelin expression/mm^2^, and then exported to SPSS (V21, SPSS Inc., IL, USA) for statistical analyses. All values are expressed as mean ± standard error of the mean (SEM). Student’s *t*-test was used when comparing reelin expression between the paired groups of dorsal to ventral hippocampi, saline and nicotine, and IHH and air (combining the 1 and 4 day piglets to determine the overall effect). Subgroup analysis to compare the individual effects of 1D IHH and 4D IHH to their corresponding control groups was performed via ANOVA using post hoc Tukey. A *p*-value ≤ 0.05 was considered statistically significant.

## Results

### Piglet Characteristics

A total of 27 piglets were included in this dataset and belonged to one of the 6 exposure groups. The piglets were derived from a total of 18 litters all mixed; thus, each exposure group was made up of a minimum of 2 litters and maximum of 5. All piglet characteristics are reported in Table 1. Piglets were male and aged between 13 and 14 postnatal days. Brain weight at death was significantly higher in the 4D air control compared to 4D IHH (*p* = 0.04, Table 1). No parameter differed in piglets exposed to nicotine compared to their respective controls (Table 1) with the exception of cotinine levels (average of 129.8 ± 35.5 ng/mL in the serum and 471.0 ± 279.1 ng/mL in the urine). These levels are consistent with those reported in infants who were either breastfeed from cigarette smoking mothers and from passive smoke exposure households (Jarvis et al. 1984; Luck and Nau 1985).

**Table 1:** Piglet characteristics between exposure groups and their corresponding controls

**Piglet Exposure Groups**								
**Characteristics**	**Saline controls** ***(n* = *5)***	**Nicotine** ***(n* = *5)***	**Air controls *(n* = *7)***	**IHH** ***(n* = *10)***	**1D air control** ***(n* = *4)***	**1D IHH** ***(n* = *6)***	**4D air control** ***(n* = *3)***	**4D IHH** ***(n* = *4)***
Age at Euthanasia *(days)*	13.4 ± 0.2	13.4 ± 0.2	14.0 ± 0.0	13.8 ± 0.1	14.0 ± 0.0	13.8 ± 0.2	14.0 ± 0.0	13.8 ± 0.3
Body weight at death *(kg)*	1.9 ± 0.5	2.0 ± 0.3	2.6 ± 0.2	2.1 ± 0.2	2.5 ± 0.3	2.2 ± 0.2	2.6 ± 0.2	2.1 ± 0.2
Brain weight at death *(g)*	37.9 ± 1.2	36.2 ± 0.7	42.0 ± 1.3	39.2 ± 0.6	40.3 ± 1.9	39.6 ± 0.3	44.3 ± 0.3	38.6 ± 1.5*
Brain/body weight ratio	2.6 ± 0.6	2.0 ± 0.3	1.7 ± 0.1	1.9 ± 0.2	1.7 ± 0.2	2.0 ± 0.3	1.7 ± 0.1	1.9 ± 0.1

Data presented as mean ± SEM

^*^ indicates statistical significance (*p* < 0.055) when compared to 4D air control following ANOVA with post hoc Tukey

### Immunostaining and Morphology

Reelin-positive cells were identified as having brown cytoplasmic staining and were seen throughout all hippocampal layers and DG (Fig. 1A–E). Cell morphology was dependent on region, with positive cells showing fusiform, circular unipolar, and bipolar shapes (Fig. 1F–H). Reelin-positive cells within the SO, SP, SR, and SLM were mostly circular and occasionally unipolar, while bipolar cells were centred around the HF (Fig. 1C, E).

Within the dorsal DG, distinct layers of the SGZ, GCL, IML, and OML were visible (Fig. 1B). Reelin expressing cells within the CA4/Hilus were larger than those in remaining layers. On occasion, it was noticed that a blood vessel would protrude through the GCL separating GC’s. When such a blood vessel was present, there was little expression of reelin in its vicinity (Fig. 2D).

Morphologically, cells of the DG GCL in the external limb were more mature than those in the internal limb (Fig. 2C). When we compare this to the reelin expression within the GCL of the external (36.7 ± 8.2 positive reelin cells/mm^2^) and internal (3.4 ± 3.4 positive reelin cells/mm^2^) limb in the control groups (*n* = 7), reelin expression was greater in the external limb (*p* = 0.004). None of the morphological features reported in human infant DG structures was observed in the ventral DG of any piglet sections in any treatment groups, whereas GCD was seen in the dorsal DG of 2 piglets: one each of a nicotine and saline group (Fig. 2A, B).

### Reelin in the Dorsal vs Ventral Hippocampus and Amongst the Layers

Comparing the dorsal to the ventral hippocampi, the dorsal hippocampus was divided into two separate blades, while the ventral adopted the well-known C shape (Fig. 1A). Reelin expression within the dorsal DG was on either the inner or outer side of the GCL whereas reelin was distributed throughout the ventral GCL. The dorsal GCL contained more layering of granule cells, creating a more tightly packed appearance, and when measured was wider (0.08 mm ± 0.00) than the ventral GCL (0.06 mm ± 0.01) DG (*p* < 0.01) (Fig. 1B vs C).

We found no differences in reelin expression in the dorsal and ventral DG between the nicotine group (*n* = 3) and their respective controls (*n* = 3), so the groups were combined for regional analyses. Reelin expression in the ventral DG was highest in the GCL followed by the SGZ (Table 2) while in the dorsal DG, this was reversed with highest levels in the SGZ (*p* = 0.005; Table 2).

**Table 2:** Comparison of reelin expression between ventral and dorsal hippocampi

**Region**	**Ventral** ***(n* = *6)***	**Dorsal** ***(n* = *6)***	***p*-value**
***Dentate gyrus***			
• Subgranular zone	96 ± 15	213 ± 29	**<0.01**
• Granule cell layer	130 ± 26	73 ± 18	0.10
• Inner molecular layer	45 ± 9	27 ± 8	0.16
• Outer molecular layer	68 ± 11	70 ± 6	0.89
Sum	340 ± 43	383 ± 39	0.48
***Cornu Ammonis 4/Hilus***	68 ± 9	96 ± 14	0.12

Data presented as mean number of positive reelin cells/mm^2^ ± SEM. *p*-values are for comparing the means. Bold indicates statistical significance, which was taken at *p* < 0.05

**Fig. 3:**
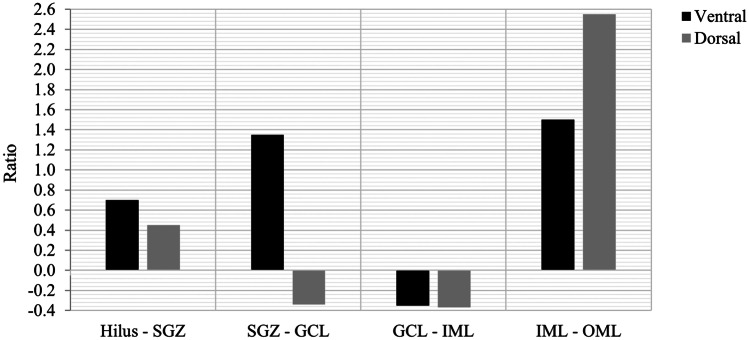
Ratios used to represent reelin migration in the subregions of the DG and CA4/Hilus in the dorsal and ventral hippocampi. A negative ratio was taken to indicate less frequent reelin migration from one layer to the next

As a proxy for reelin “migration”, we compared reelin expression in each region to that in the adjacent region (Fig. 3). Reelin cells in the ventral DG appear to have migrated to adjacent regions more frequently than the dorsal. In the dorsal DG, reelin migration from the SGZ to the GCL and GCL to IML appeared less frequent (Fig. 3).

### Effects of Nicotine Exposure

There was no clear effect when comparing the nicotine to saline controls apart from a trend to increased reelin in the IML (*p* = 0.06, Table 3, Supplementary Table 1).

**Table 3:** Effects of nicotine exposure on reelin expression in the piglet hippocampus

**Region**	**Saline controls** ***(n* = *5)***	**Nicotine** ***(n* = *5)***	***p*-value**
***Dentate gyrus***			
• Subgranular zone	89 ± 15	112 ± 24	0.43
• Granule cell layer	160 ± 45	156 ± 36	0.94
• Inner molecular layer	28 ± 6	53 ± 9	*0.06*
• Outer molecular layer	64 ± 14	55 ± 8	0.60
Sum^**a**^	342 ± 60	376 ± 62	0.70
***Cornu Ammonis 1***			
• Stratum oriens	90 ± 17	74 ± 11	0.43
• Stratum pyramidale	68 ± 21	38 ± 13	0.25
• Stratum radiatum	75 ± 12	88 ± 23	0.63
• Stratum lacunosum moleculare	73 ± 12	102 ± 32	0.42
• Hippocampal fissure	214 ± 41	168 ± 33	0.41
Sum^**a**^	520 ± 70	470 ± 77	0.64
***Cornu Ammonis 4/Hilus***^**a**^	78 ± 10	82 ± 19	0.88

Data presented as mean number of positive reelin cells/mm^2^ ± SEM

Student *T*-test was applied to compare the nicotine to saline. Bold indicates statistical significance, taken at *p* < 0.05. Italics indicate a trending statistical significance between *p* = 0.05 and 0.09

^a^Box plots showing individual data points, as well as median and range are provided in Supplementary Fig. 1

### Effects of IHH Exposure

The overall effect of IHH exposure was a decrease in reelin in the CA1 (*p* = 0.05, Table 4, Supplementary Table 2), specifically in the layers of the SP (*p* = 0.04, Table 4) and HF (*p* = 0.02, Table 4).

**Table 4:** Effects of IHH exposure on reelin expression in the piglet hippocampus, as a whole group and separated according to duration

**Region**	**Air control** **(*n* = 7)**	**IHH** **(*n* = 10)**	***p*-value**	**1D air control** ***(n* = *4)***	**1D IHH** ***(n* = *6)***	***p*-value**	**4D air control** ***(n* = *3)***	**4D IHH** ***(n* = *4)***	***p*-value**
***Dentate gyrus***									
• Subgranular zone	78 ± 13	78 ± 11	1.00	63 ± 10	88 ± 14	0.64	97 ± 27	62 ± 15	0.52
• Granule cell layer	45 ± 10	72 ± 16	0.21	45 ± 11	65 ± 19	0.90	44 ± 22	83 ± 30	0.69
• Inner molecular layer	30 ± 6	26 ± 6	0.68	23 ± 10	25 ± 5	1.00	39 ± 5	27 ± 15	0.39
• Outer molecular layer	47 ± 7	34 ± 5	0.16	40 ± 11	34 ± 8	0.96	56 ± 5	34 ± 9	0.85
Sum^**a**^	199 ± 26	210 ± 27	0.79	171 ± 21	213 ± 28	0.89	237 ± 52	205 ± 58	0.96
***Cornu Ammonis 1***									
• Stratum oriens	125 ± 16	121 ± 20	0.89	103 ± 20	132 ± 21	0.85	154 ± 17	104 ± 43	0.65
• Stratum pyramidale	74 ± 14	40 ± 8	**0.04**	84 ± 18	38 ± 12	0.14	59 ± 25	44 ± 6	0.91
• Stratum radiatum	119 ± 20	98 ± 16	0.41	116 ± 30	105 ± 21	0.76	124 ± 33	87 ± 25	0.82
• Stratum lacunosum moleculare	118 ± 17	91 ± 17	0.29	109 ± 31	98 ± 27	0.99	129 ± 6	80 ± 18	0.63
• Hippocampal fissure	189 ± 27	97 ± 21	**0.02**	185 ± 50	92 ± 33	0.25	194 ± 3	104 ± 26	0.41
Sum^**a**^	625 ± 73	446 ± 47	**0.05**	597 ± 128	465 ± 57	0.66	661 ± 59	418 ± 89	0.32
***Cornu Ammonis 4/Hilus***^**a**^	73 ± 13	80 ± 11	0.68	54 ± 10	92 ± 16	0.25	100 ± 18	63 ± 9	0.42

Data presented as mean number of positive reelin cells/mm^2^ ± SEM

Student *T*-test was applied to compare the air to IHH as a whole group, and an ANOVA with Tukey post hoc to compare amongst the duration of IHH groups, with *p* values provided comparing each IHH group to its counterpart (i.e. 1D IHH vs 1D air, and 4D IHH vs 4 D air). Bold indicates statistical significance, taken at *p* < 0.05. Note, no differences were seen comparing the 1D air to 4D air, and 1D IHH to 4D IHH (Supplementary Table 3)

^a^Box plots showing individual data points, as well as median and range are provided in Supplementary Fig. 1

No differences were found in expression levels between the two air control groups (Table 4, Supplementary Table 3) nor between the 1D IHH and 4D IHH groups (Table 4, Supplementary Table 3).

## Discussion

The main findings in this study are that (i) expression of reelin in the SGZ is higher in the dorsal than the ventral DG, (ii) nicotine exposure had no significant effect on reelin expression in the hippocampus and DG, (iii) IHH exposure decreased reelin expression in the SP and HF layers of CA1, and with (iv) no additional impact on reelin expression due to duration of IHH exposure.

### Cellular Expression and Distribution of Reelin in the Piglet Hippocampus

Consistent with published literature, reelin was expressed in all layers of the CA1 and DG, and the distribution and cellular expression are similar to those seen by Ábrahám et al. (2004) in the adult domestic pig. Bipolar cells were located in the HF and perpendicular to the OML, with minimal expression in IML and CA4/Hilus (Ábrahám et al. 2004).

There do appear to be species differences in expression amongst the layers. We reported that reelin expression in the SP of the human was minimal (Despotovski et al. 2021). Moreover, in the human, reelin was predominantly located on the ML side of the ventral DG (Despotovski et al. 2021), whereas in piglets, it was in the SGZ. Reelin distribution in rodents appears more closely aligned to piglets than humans, with an abundance of reelin in the Hilus but weak expression in the ML (Borrell et al. 1999; Ramos-Moreno et al. 2006; Knuesel et al. 2009), as well as a higher reelin count within the SML (the HF included within this layer) and SO, as opposed to the SP layer in adult rats (Ramos-Moreno et al. 2006).

### Dorsal and Ventral Hippocampus

The higher reelin expression in the SGZ in the dorsal region may relate to function, or to development. The ventral hippocampus, being part of the temporal pole, is involved in motivational and emotional behaviour (Ábrahám et al. 2004; Fanselow and Dong 2010) while the dorsal hippocampus, located in the septal pole, is involved in navigation, learning, and memory (Fanselow and Dong 2010).

The SGZ is known to be a site of neurogenesis within the hippocampus (Altman and Das 1965; Brazel et al. 2003; Spalding et al. 2013; Boldrini et al. 2018). This is the first study to report on reelin expression in the SGZ. A single canine study compared levels of a variety of molecular markers between the dorsal and ventral DG and found more evidence of neurogenesis (proliferation, mature neurons, and granule cells) in the dorsal than the ventral hippocampus (Bekiari et al. 2020). That study also reported that migration of newborn granule cells from the SGZ to the GCL was faster in the dorsal than the ventral region (Bekiari et al. 2020), similar to our result here in piglets (Fig. 3). Combined, we theorise that the higher expression of reelin is linked to the higher rate of neurogenesis in the dorsal than the ventral region, thus adding to the literature the importance of studies specifying the exact region being analysed.

The role of reelin in neuronal migration within the DG has been best studied in hippocampal cell cultures. Wang et al. (2018) confirmed that reelin exerts an attractive effect within the DG and controls the direction that granule cells migrate, but not their migratory process or speed. In wild-type mice, granule cells from the Hilus migrate towards the GCL leading to a well-compacted GCL. In reelin-deficient mice, granule cell migration is less directed, does not form a compact GCL, and granule cells often invade the IML (Wang et al. 2018; Zhao et al. 2004)). Our data also suggests that migration is from the Hilus/CA4 to the SGZ in the young piglet (Fig. 3).

### Effects of Nicotine

Cigarette smoke exposure in pregnancy is one of the most modifiable risks for morbidity to the infant (Blood-Siegfried and Rende 2010). Chronic neonatal exposure to cigarette smoke affects cell morphology in the hippocampus (Roy and Sabherwal 1998) and increases cell death (Huang et al. 2007). Nicotine exposure also affects hippocampal neurogenesis by inducing a decrease in markers of proliferation and migration including BrdU and Polysialic acid-neural cell adhesion molecule (PSA-NCAM) cells, and an increase in pyknotic cells (Abrous et al. 2002). Studies of the effect of nicotine exposure on reelin expression within the hippocampus are limited. Three studies in rodents using PCR on hippocampal homogenates (Romano et al. 2013a, b), and histochemical analysis in the Hilus of Sprague–Dawley rats (Ohishi et al. 2014), reported no significant difference in reelin expression after exposure to nicotine. Our findings are consistent with these, strengthening the data of a lack of effect of nicotine on reelin expression in the hippocampus.

### Effects of Hypercapnic Hypoxia

As previously mentioned, most animal studies show that exposure to hypoxia is associated with decreased reelin expression in the brain (Golan et al. 2009; Haramati et al. 2010; Komitova et al. 2013; Howell and Pillai 2014, 2016; Nisimov et al. 2018). We incorporated hypercapnia in our model to more closely mimic the physiology of conditions of obstructive sleep apnea (OSA) (Waters and Tinworth 2001) and risk factors of SIDS such as prone-sleeping. We reported within this study a significant decrease of reelin expression in the CA1 after exposure of IHH, consistent with previous homogenate studies (Golan et al. 2009; Howell and Pillai 2014). In addition, we located a specific regional decrease within the layers of SP and HF, and found no impact attributable to duration of exposure to IHH.

Our localisation of the finding to the HF and SP layers is novel. The HF is the site of interface that separates the SLM layer of the CA1 from the ML of the DG. The entorhinal cortex (EC) projects fibers through the perforant path and these are crossed through the HF (Ramón 1909-11). Studies show that more than 75% of reelin expressing CR cells are located within the HF (Abraham and Meyer 2003), and that in the human hippocampus, it had high reelin expression (Despotovski et al. 2021), as it did herein within our piglets. The SP layer transmits its axons to the subiculum and EC (Graves et al. 2013), and is reported to be highly sensitive in acute and chronic hypoxic exposures whereby cell density is significantly decreased (Mikati et al. 2005). Combined, it is feasible that reduced reelin expression affects cell density in the SP, with a likely assumption being altered migration from the HF, but future stereological studies would be required to support this hypothesis.

## Conclusion

This is the first study to report on regional reelin expression in the young piglet hippocampal CA1 and DG. We found reelin expressing cells in all layers of CA1 and DG, with greater expression in the dorsal than ventral regions. While nicotine exposure did not affect reelin expression, IHH was associated with decreased reelin expression in the SP and HF layers of CA1, independent of the duration of IHH exposure. We postulate that reduced reelin expression after hypercapnic hypoxic exposures could translate to reduced cell migration.

## Supplementary Information

Below is the link to the electronic supplementary material.

Supplementary file1 (DOCX 288 kb)

## Data Availability

The datasets generated and analysed in the current study are available from the corresponding author upon reasonable request.

## Abbreviations

dH_2_O: Distilled H_2_O
IHC: Immunohistochemistry
NHS: Normal horse serum
PBS: Phosphate buffered saline
PMI: Post-mortem interval
SEM: Standard error of the mean
IHH: Intermittent hypercapnic hypoxia
GC: Granule cell
SGZ: Subgranular zone
GCL: Granule cell layer
DG: Dentate gyrus
IML: Inner molecular layer
OML: Outer molecular layer
HF: Hippocampal fissure
SLM: Stratum lacunosum moleculare
SR: Stratum radiatum
SP: Stratum pyramidale
SO: Stratum oriens
CR: Cajal-Retzius
CA: Cornu amonis
