# Supplementary material for: Early Postnatal Exposure to Intermittent Hypercapnic Hypoxia (IHH), but Not Nicotine, Decreases Reelin in the Young Piglet Hippocampus
Source: Neurotox Res. 2022 Nov 2;40(6):1859–68. doi: 10.1007/s12640-022-00598-0 (PMC9797456; doi:10.1007/s12640-022-00598-0)
Supplement: Supplementary file 1 — Supplementary file1 (DOCX 288 kb) [file 12640_2022_598_MOESM1_ESM.docx]

**Supplementary Section**

**Supplementary Table 1: Comparison of Nicotine (n=5) and Saline (n=5) groups using Student T-Test.**

| **Independent Samples Test** | | | | | | | | | | |
| --- | --- | --- | --- | --- | --- | --- | --- | --- | --- | --- |
|  | | Levene's Test for Equality of Variances | | t-test for Equality of Means | | | | | | |
|  |  | F | Sig. | t | df | Sig. (2-tailed) | Mean Difference | Std. Error Difference | 95% Confidence Interval of the Difference | |
|  |  |  |  |  |  |  |  |  | Lower | Upper |
| DG | Equal variances assumed | .000 | .991 | -.399 | 8 | .700 | -34.293 | 85.966 | -232.532 | 163.944 |
|  | Equal variances not assumed |  |  | -.399 | 7.994 | .700 | -34.293 | 85.966 | -232.557 | 163.969 |
| GCL | Equal variances assumed | .807 | .395 | .076 | 8 | .941 | 4.365 | 57.351 | -127.888 | 136.619 |
|  | Equal variances not assumed |  |  | .076 | 7.676 | .941 | 4.365 | 57.351 | -128.866 | 137.596 |
| IML | Equal variances assumed | 1.618 | .239 | -2.211 | 8 | .058 | -24.112 | 10.908 | -49.267 | 1.041 |
|  | Equal variances not assumed |  |  | -2.211 | 6.590 | .065 | -24.112 | 10.908 | -50.235 | 2.009 |
| OML | Equal variances assumed | 4.272 | .073 | .540 | 8 | .604 | 8.653 | 16.029 | -28.309 | 45.616 |
|  | Equal variances not assumed |  |  | .540 | 6.171 | .608 | 8.653 | 16.029 | -30.306 | 47.613 |
| SGZ | Equal variances assumed | 3.667 | .092 | -.831 | 8 | .430 | -23.199 | 27.924 | -87.593 | 41.194 |
|  | Equal variances not assumed |  |  | -.831 | 6.812 | .434 | -23.199 | 27.924 | -89.601 | 43.202 |
| CA4/HILUS | Equal variances assumed | 6.179 | .038 | -.151 | 8 | .884 | -3.271 | 21.667 | -53.235 | 46.693 |
|  | Equal variances not assumed |  |  | -.151 | 5.972 | .885 | -3.271 | 21.667 | -56.348 | 49.805 |
| CA1 | Equal variances assumed | .422 | .534 | .486 | 8 | .640 | 50.560 | 104.038 | -189.352 | 290.473 |
|  | Equal variances not assumed |  |  | .486 | 7.936 | .640 | 50.560 | 104.038 | -189.689 | 290.809 |
| SO | Equal variances assumed | 1.848 | .211 | .815 | 8 | .439 | 16.427 | 20.155 | -30.050 | 62.905 |
|  | Equal variances not assumed |  |  | .815 | 6.665 | .443 | 16.427 | 20.155 | -31.722 | 64.577 |
| SP | Equal variances assumed | .342 | .575 | 1.226 | 8 | .255 | 30.616 | 24.974 | -26.975 | 88.209 |
|  | Equal variances not assumed |  |  | 1.226 | 6.602 | .262 | 30.616 | 24.974 | -29.168 | 90.402 |
| SR | Equal variances assumed | .957 | .357 | -.495 | 8 | .634 | -12.791 | 25.855 | -72.414 | 46.831 |
|  | Equal variances not assumed |  |  | -.495 | 6.073 | .638 | -12.791 | 25.855 | -75.873 | 50.290 |
| SLM | Equal variances assumed | 6.634 | .033 | -.846 | 8 | .422 | -29.006 | 34.286 | -108.071 | 50.059 |
|  | Equal variances not assumed |  |  | -.846 | 5.066 | .436 | -29.006 | 34.286 | -116.800 | 58.788 |
| HF | Equal variances assumed | .323 | .586 | .869 | 8 | .410 | 45.312 | 52.172 | -74.996 | 165.621 |
|  | Equal variances not assumed |  |  | .869 | 7.682 | .411 | 45.312 | 52.172 | -75.868 | 166.493 |

**Supplementary Table 2: Comparison of IHH (n=10) and Air (n=7) groups using Student T-Test.**

| **Independent Samples Test** | | | | | | | | | | | |
| --- | --- | --- | --- | --- | --- | --- | --- | --- | --- | --- | --- |
|  | | Levene's Test for Equality of Variances | | t-test for Equality of Means | | | | | | | |
|  |  | F | Sig. | t | df | Sig. (2-tailed) | Mean Difference | Std. Error Difference | 95% Confidence Interval of the Difference | |  |
|  |  |  |  |  |  |  |  |  | Lower | Upper |  |
| DG | Equal variances assumed | .483 | .498 | -.272 | 15 | .789 | -10.565 | 38.782 | -93.229 | 72.097 |  |
|  | Equal variances not assumed |  |  | -.282 | 14.494 | .782 | -10.565 | 37.402 | -90.529 | 69.398 |  |
| GCL | Equal variances assumed | 3.908 | .067 | -1.303 | 15 | .212 | -27.301 | 20.959 | -71.976 | 17.3727 |  |
|  | Equal variances not assumed |  |  | -1.449 | 14.190 | .169 | -27.301 | 18.843 | -67.666 | 13.0627 |  |
| IML | Equal variances assumed | .042 | .841 | .426 | 15 | .676 | 3.796 | 8.913 | -15.201 | 22.794 |  |
|  | Equal variances not assumed |  |  | .440 | 14.375 | .666 | 3.796 | 8.629 | -14.665 | 22.2598 |  |
| OML | Equal variances assumed | .101 | .756 | 1.492 | 15 | .157 | 12.992 | 8.711 | -5.574 | 31.560 |  |
|  | Equal variances not assumed |  |  | 1.470 | 12.394 | .166 | 12.992 | 8.837 | -6.193 | 32.179 |  |
| SGZ | Equal variances assumed | .162 | .693 | -.003 | 15 | .998 | -.053 | 17.100 | -36.502 | 36.395 |  |
|  | Equal variances not assumed |  |  | -.003 | 12.843 | .998 | -.053 | 17.184 | -37.224 | 37.116 |  |
| CA4/HILUS | Equal variances assumed | .441 | .517 | -.420 | 15 | .680 | -7.047 | 16.773 | -42.800 | 28.705 |  |
|  | Equal variances not assumed |  |  | -.423 | 13.389 | .679 | -7.047 | 16.655 | -42.923 | 28.829 |  |
| CA1 | Equal variances assumed | .014 | .907 | 2.162 | 15 | .047 | 178.194 | 82.413 | 2.533 | 353.855 |  |
|  | Equal variances not assumed |  |  | 2.058 | 10.722 | .065 | 178.194 | 86.593 | -13.001 | 369.390 |  |
| SO | Equal variances assumed | 1.771 | .203 | .147 | 15 | .885 | 4.108 | 27.863 | -55.280 | 63.497 |  |
|  | Equal variances not assumed |  |  | .158 | 14.995 | .876 | 4.108 | 25.945 | -51.193 | 59.411 |  |
| SP | Equal variances assumed | 1.107 | .309 | 2.242 | 15 | .040 | 33.392 | 14.893 | 1.648 | 65.137 |  |
|  | Equal variances not assumed |  |  | 2.065 | 9.308 | .068 | 33.392 | 16.167 | -2.997 | 69.783 |  |
| SR | Equal variances assumed | .004 | .952 | .848 | 15 | .410 | 21.391 | 25.232 | -32.390 | 75.172 |  |
|  | Equal variances not assumed |  |  | .833 | 12.267 | .421 | 21.391 | 25.664 | -34.392 | 77.174 |  |
| SLM | Equal variances assumed | .611 | .447 | 1.087 | 15 | .294 | 27.136 | 24.974 | -26.095 | 80.368 |  |
|  | Equal variances not assumed |  |  | 1.122 | 14.354 | .280 | 27.136 | 24.194 | -24.634 | 78.907 |  |
| HF | Equal variances assumed | .124 | .730 | 2.726 | 15 | .016 | 92.164 | 33.812 | 20.095 | 164.234 |  |
|  | Equal variances not assumed |  |  | 2.703 | 12.679 | .018 | 92.164 | 34.096 | 18.313 | 166.015 |  |

**Supplementary Table 3: Comparison of 1D IHH, 1D Air, 4D IHH and 4D Air groups using ANOVA Tukey.**

| **ANOVA** | | | | | | |
| --- | --- | --- | --- | --- | --- | --- |
|  | | Sum of Squares | df | Mean Square | F | Sig. |
| DG | Between Groups | 7906.075 | 3 | 2635.358 | .401 | .755 |
|  | Within Groups | 85453.723 | 13 | 6573.363 |  |  |
|  | Total | 93359.798 | 16 |  |  |  |
| GCL | Between Groups | 3802.773 | 3 | 1267.591 | .624 | .612 |
|  | Within Groups | 26400.102 | 13 | 2030.777 |  |  |
|  | Total | 30202.875 | 16 |  |  |  |
| IML | Between Groups | 477.837 | 3 | 159.279 | .461 | .714 |
|  | Within Groups | 4488.458 | 13 | 345.266 |  |  |
|  | Total | 4966.295 | 16 |  |  |  |
| OML | Between Groups | 1149.322 | 3 | 383.107 | 1.177 | .357 |
|  | Within Groups | 4232.725 | 13 | 325.594 |  |  |
|  | Total | 5382.048 | 16 |  |  |  |
| SGZ | Between Groups | 3740.897 | 3 | 1246.966 | 1.132 | .372 |
|  | Within Groups | 14320.670 | 13 | 1101.590 |  |  |
|  | Total | 18061.567 | 16 |  |  |  |
| CA4/Hilus | Between Groups | 5780.214 | 3 | 1926.738 | 2.122 | .147 |
|  | Within Groups | 11802.788 | 13 | 907.907 |  |  |
|  | Total | 17583.003 | 16 |  |  |  |
| CA1 | Between Groups | 143025.124 | 3 | 47675.041 | 1.522 | .256 |
|  | Within Groups | 407232.651 | 13 | 31325.589 |  |  |
|  | Total | 550257.775 | 16 |  |  |  |
| SO | Between Groups | 6631.628 | 3 | 2210.543 | .694 | .572 |
|  | Within Groups | 41389.381 | 13 | 3183.799 |  |  |
|  | Total | 48021.009 | 16 |  |  |  |
| SP | Between Groups | 5741.890 | 3 | 1913.963 | 1.983 | .166 |
|  | Within Groups | 12549.858 | 13 | 965.374 |  |  |
|  | Total | 18291.748 | 16 |  |  |  |
| SR | Between Groups | 2772.473 | 3 | 924.158 | .313 | .816 |
|  | Within Groups | 38435.109 | 13 | 2956.547 |  |  |
|  | Total | 41207.582 | 16 |  |  |  |
| SLM | Between Groups | 4539.282 | 3 | 1513.094 | .531 | .669 |
|  | Within Groups | 37016.889 | 13 | 2847.453 |  |  |
|  | Total | 41556.171 | 16 |  |  |  |
| HF | Between Groups | 35453.937 | 3 | 11817.979 | 2.190 | .138 |
|  | Within Groups | 70137.207 | 13 | 5395.170 |  |  |
|  | Total | 105591.145 | 16 |  |  |  |

|  | **Mean with SEM** | **Median with Interquartile Range** |
| --- | --- | --- |
| **DG** | 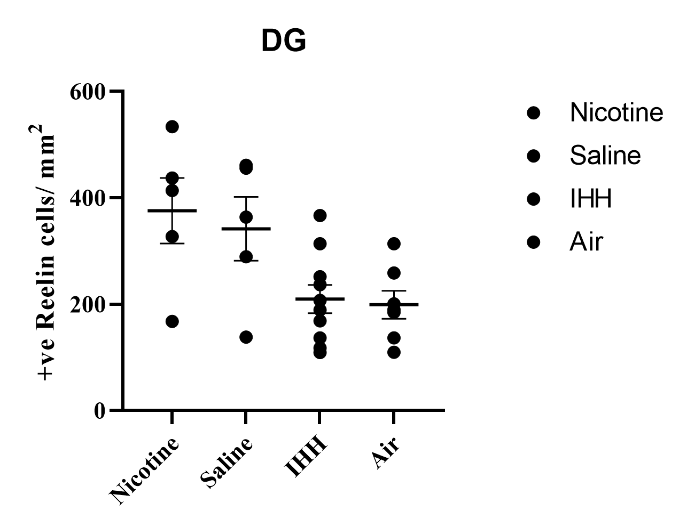 | 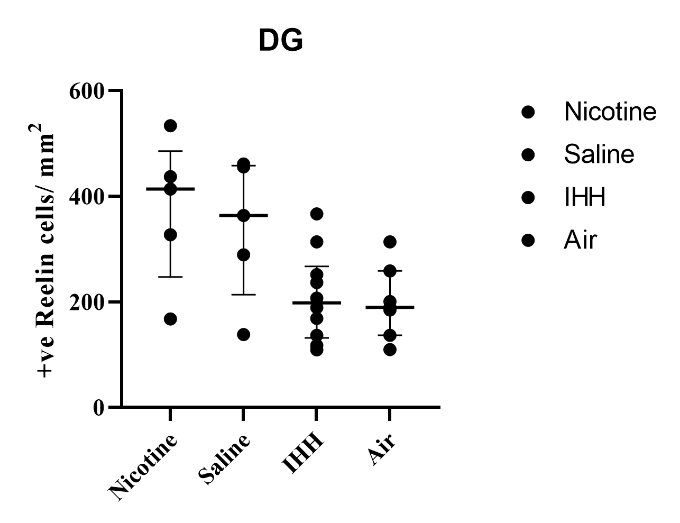 |
| **CA4/Hilus** | 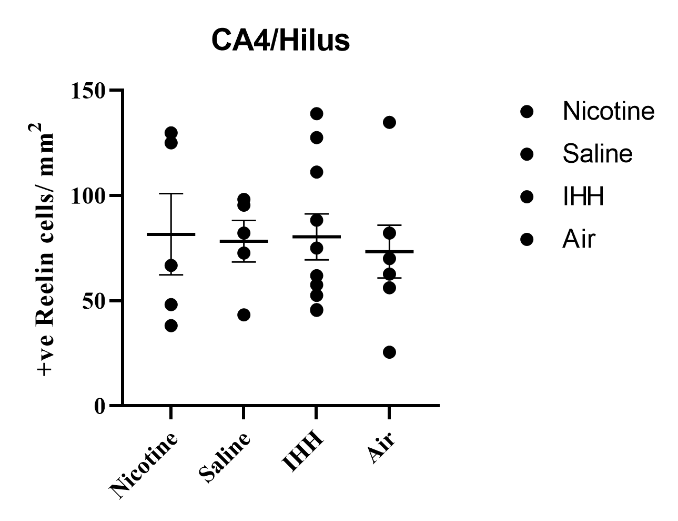 | 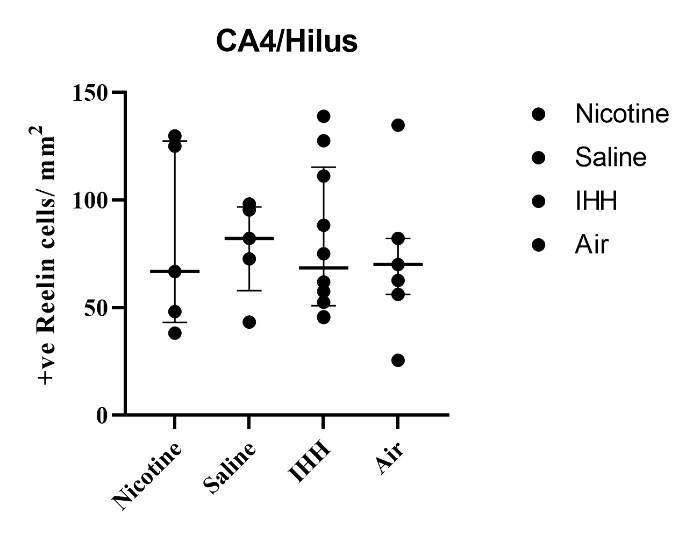 |
| **CA1** | 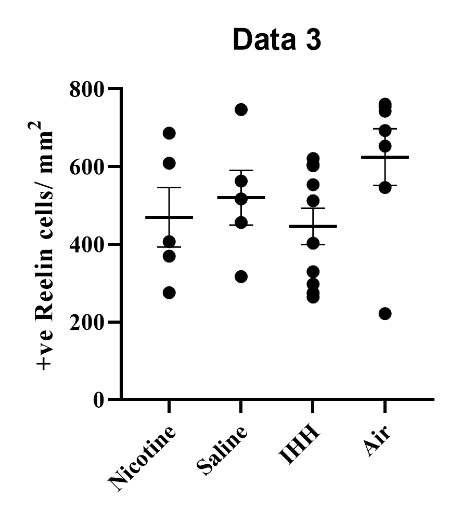 | 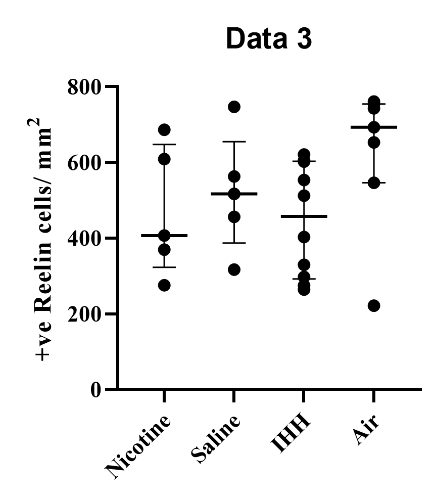 |

**Supplementary Figure 1**: Sum of DG, CA4/Hilus and CA1 for Nicotine (n=5), Saline (n=5), IHH (n=10) and Air (n=7) treatment groups presented as boxplots in the form of Mean with SEM and Median with Interquartile Range.
